# Getting to grips with c-Myc

**DOI:** 10.7554/eLife.32010

**Published:** 2018-01-09

**Authors:** Dirk Eick

**Affiliations:** 1 Department of Molecular EpigeneticsHelmholtz Center MunichMunichGermany; 2 Center for Integrated Protein Science Munich (CIPSM)MunichGermany

**Keywords:** Reproducibility Project: Cancer Biology, replication, metascience, reproducibility, c-Myc, gene expression, Human

## Abstract

The transcription factor c-Myc amplifies the transcription of many growth-related genes in cancer cells, but its role as an oncogene is not fully understood.

**Related research article** Lewis LM, Edwards MC, Meyers ZR, Talbot Jr CC, Hao H, Blum D, Reproducibility Project: Cancer Biology. 2018. Replication Study: Transcriptional amplification in tumor cells with elevated c-Myc. *eLife* **7**:e30274. doi: 10.7554/eLife.30274

c-Myc is a transcription factor that is involved in a number of cellular processes. It is also known that high levels of c-Myc increase the likelihood of normal cells developing into tumor cells. However, several key questions about the role c-Myc in both normal and cancer cells remain unanswered.

In 2012 researchers at the Whitehead Institute for Biomedical Research and a number of other institutes in Cambridge and Boston reported that, in human tumor cells expressing high levels of c-Myc, the transcription factor accumulates in the promoter regions of active growth-related genes, which leads to 'transcriptional amplification' (Lin et al., 2012). In other words, rather than increasing the transcription of a specific set of target genes, c-Myc increases the transcription of all active genes. At the same time an independent group, led by researchers at the National Institutes of Health, reported that c-Myc also leads to 'transcriptional amplification' in lymphocytes and embryonic stem cells taken from mice (Nie et al., 2012).

Lin et al. investigated the human P493-6 B cell model of Burkitt's lymphoma: these cells do not naturally express c-Myc, but they contain a c-Myc transgene that is regulated by a chemical called tetracycline (Schuhmacher et al., 1999). Using Affymetrix microarray-based technology to study all the mRNA transcripts produced by the P493-6 cells in the presence of low and high levels of c-Myc, Lin et al. came to two major conclusions. First, c-Myc acts primarily to stimulate transcription in P493-6; the repression of transcription was seen in only a very small number of genes. Second, silent genes – genes that are expressed at very low levels or not at all – were generally not activated by elevated c-Myc levels.

Subsequent work by other researchers also explored the role of c-Myc in cancer cells. In 2013 the present author reported that c-Myc can induce and amplify the transcription of some 447 previously reported target genes in a dose-dependent manner (Schuhmacher and Eick, 2013), and in 2014 two independent groups reported that c-Myc can repress active genes and, moreover, that the amplification effect it exerts on all genes is the result of it up-regulating a specific set of target genes (Sabò et al., 2014; Walz et al., 2014; see also Dang, 2014). Older work had also shown that the genes for ribosomal RNA were probably the most important targets of c-Myc, and that the resulting increase in ribosomal RNA led to an significant increase in the total number of RNA transcripts per cell (Arabi et al., 2005; Grandori et al., 2005).

In 2015, as part of the Reproducibility Project: Cancer Biology, Blum et al. published a Registered Report which explained in detail how they would seek to replicate selected experiments from the paper by Lin et al. (Blum et al., 2015). The results of these experiments have now been published as a Replication Study (Lewis et al., 2018). This study largely confirms the results of the original study by Lin et al., but it also reveals that results can be strongly influenced by biological variables and by the statistical methods used to analyze the data.

The P493-6 cells used for the Replication Study were obtained from the team at the Whitehead Institute, but the batch of serum that was used to cultivate the cells in the original experiments was no longer available, so the Replication Study was performed with two new lots of serum. While the new lots of serum did not affect the induction of high levels of c-Myc in the cells, they had a clear affect on the steady-state levels of RNA that were measured in the cells 24 hours later. In the original study of Lin et al., the steady-state RNA levels per cell increased 1.29-fold after c-Myc induction; in the Replication Study a 1.14-fold increase was seen for serum lot 1, and a 1.43-fold increase was seen for serum lot 2. However, this variation was not unexpected because a previous study had shown that serum has an influence on the regulation of transcription by c-Myc in P493-6 cells (Schlosser et al., 2005).

Despite the influence exerted by the different lots of serum, the main finding of Lin et al. – that c-Myc acts as an amplifier for the expression of active genes in P493-6 cells – could be confirmed. Virtually the same set of genes that was up-regulated in response to c-Myc in the original study was also up-regulated in the Replication Study for serum lot 2. The up-regulation of various genes was also observed for serum lot 1, but the data were not statistically significant because the response of the genes to c-Myc was weak.

The Replication Study further revealed that the statistical methods used to analyze the data can influence the interpretation of the results. In the original study genes were classified as 'silent' if there was less than 0.5 mRNA transcript per cell, and as 'active' if there was more than one transcript per cell. (Cells with between 0.5 and 1 transcript per cell were not included in the analysis). Lin et al. reported that there was a significant increase in the transcription of active genes, but not silent genes, after the induction of c-Myc. Unfortunately, separating the genes into two groups like this, a process termed dichotomization, can lead to a loss of information in the subsequent analysis (Altman and Royston, 2006). When the data from the original study were re-analyzed as paired samples, it became evident that silent genes were significantly up-regulated after c-Myc induction. A similar effect was observed when the new data were analyzed as paired samples: however, the number of silent genes that showed significant activation was much lower than the number of active genes that were amplified.

It is clear that c-Myc amplifies the transcription of many growth-related genes in P493-6 cells, as reported by Lin et al. (and also by Nie et al.). Together with the elevated levels of ribosomal RNA (see above), the amplification of transcripts of RNA polymerase I and the amplification of Pol II transcripts of c-Myc target genes can explain the increase in the levels of RNA measured in cells 24 hours after the induction of c-Myc. It remains to be investigated if and how the activation of silent genes further contributes to c-Myc's function as an oncogene.

## Note

Dirk Eick was one of the reviewers for the Registered Report (Blum et al., 2015) and the Replication Study (Lewis et al., 2018).
